# A Landscape View of the Female Genital Tract Microbiome in Healthy Controls and Women With Reproductive Health Conditions Associated With Ectopic Pregnancy

**DOI:** 10.3389/bjbs.2023.12098

**Published:** 2024-01-12

**Authors:** Hui En Teh, Cheng Khee Pung, Valliammai Jayanthi Thirunavuk Arasoo, Polly Soo Xi Yap

**Affiliations:** Jeffrey Cheah School of Medicine and Health Sciences, Monash University Malaysia, Bandar Sunway, Selangor, Malaysia

**Keywords:** 16S rRNA, ectopic pregnancy, fallopian tube, microbiome, female genital

## Abstract

Disruption of the female genital microbiome is associated with several pregnancy complications, including miscarriage, preterm onset of labour, and tubal pregnancy. Ectopic pregnancy is a known cause of maternal morbidity and mortality, but early diagnosis and treatment of ectopic pregnancy remain a challenge. Despite growing established associations between genital microbiome and female reproductive health, few studies have specifically focused on its link with ectopic pregnancy. Therefore, the current review aims to provide a comprehensive account of the female genital microbiome in healthy and fertile women compared to those in ectopic pregnancy and its associated risk factors. The microbial diversity from various sites of the female genital tract was explored for a reliable proxy of female reproductive health in sequencing-based ectopic pregnancy research. Our report confirmed the predominance of *Lactobacillus* in the vagina and the cervix among healthy women. The relative abundance decreased in the vaginal and cervical microbiome in the disease state. In contrast, there were inconsistent findings on the uterine microbiome across studies. Additionally, we explore a spectrum of opportunities to enhance our understanding of the female genital tract microbiome and reproductive conditions. In conclusion, this study identifies gaps within the field and emphasises the need for visionary solutions in metagenomic tools for the early detection of ectopic pregnancy and other gynaecological diseases.

## Introduction

The female genital tract can be separated into the upper genital tract, which comprises the ovaries, fallopian tubes, endometrium, and cervix, and the lower genital tract, which is made up of the vulva and the vagina [1]. Generally, it has been agreed upon that the vagina is colonised by a wide range of bacteria but is physiologically dominated by *Lactobacillus.* [2] In contrast, the fallopian tubes and endometrium have classically been described as sterile sites, protected by cervical mucus, which acts as a barrier to the ascent of bacteria into the uterus [3]. However, this notion has been challenged, as it has been shown that particles can be transported from the vagina to the upper genital tract during the follicular and luteal phases of the menstrual cycle [4].

Prior to 2007, characterisation of the female genital tract was mostly done by conventional culture methods. However, this was gradually taken over by next-generation sequencing (NGS), such as 16S rRNA gene sequencing [5]. The 16S rRNA gene, also known as 16S rDNA, is the part of the DNA most commonly used for the purpose of taxonomic classification of bacteria. This method works well for samples contaminated by host DNA and low biomass samples, such as the upper genital tract [6]. Although the majority of primary research studies characterising female reproductive tract microbiome focused on the vagina due to its acceptability and ease of sampling [2, 7, 8], a number of studies investigated the cervical microbiome, with scant and fragmented evidence on the microbiome above the cervix [9].

Due to various limitations, little research has been done on the microbiome of the female genital tract in ectopic pregnancy. Researchers postulate that endometrial microbiota may play a role in the pathogenesis of ectopic pregnancy [10]. With existing knowledge, imbalances of endometrial microbiota have been associated with endometriosis, infertility, and recurrent pregnancy loss [5, 11]. Some widely explored risk factors for ectopic pregnancy include recurrent ectopic pregnancy [12], pelvic inflammatory disease [13], endometriosis, and adenomyosis [14]. In this review, we explored the similarities in these conditions and or risk factors associated with ectopic pregnancy, the changes in relative abundances of the microbiome, and the changes in diversity compared to the microbiome of healthy, fertile women. With gathered evidence, reliable proxies for potential early diagnosis and disease management in ectopic pregnancy are also discussed.

## Diversity of the Female Genital Tract Microbiome in Healthy Women

PubMed, Scopus and Ovid MEDLINE databases were used and manually screened by title, abstract, and full text for relevance at the same time, noting the inclusion and exclusion criteria. Only women of reproductive age were recruited whilst studies that recruited women who used hormonal contraceptives were excluded. Of the 31 studies selected for this review (Supplementary Material), 15 were from China, 4 from Italy, 2 from Japan, and 1 each from Australia, Spain, Germany, Turkey, Puerto Rico, Korea, United States, Taiwan, Sweden, and Thailand. The sample sizes ranged from 5 to 160 participants. Some studies investigated the microbiome of more than one genital site. Two studies analysed the microbiome of the fallopian tube, three looked at the endometrium, eight focused on the cervix, and twenty-four studies described the microbiome of the vagina. Some studies provided the mean relative abundance in percentage of the top 10-20 taxa, while some only arranged the taxa identified in order of decreasing abundance. Table 1 summarises the findings from the 31 studies included. Healthy, fertile controls from studies that characterised the microbiome in women with reproductive health conditions and in infertile women were also included, provided they were not pregnant, not using any hormonal contraception, and were not pre-menopausal. At the level of phyla, the microbiome of the female genital tract in healthy, fertile women is composed mainly of Actinobacteria, Bacteroidetes, Firmicutes, Fusobacteria, and Proteobacteria, with few studies identifying Tenericutes. Acidobacteria, Chlamydiae, Chlorofexi, Planctomycetes, and Verrucomicrobia were only identified in one study. The microbiome is also not consistent throughout the female genital tract, with variations between the fallopian tube, endometrium, cervix, and vagina.

**TABLE 1:** Summary of the diversity of the female genital tract microbiome in healthy women.

Study	Sample size	Country	Sample type	Sequencing techniques (Target region)	Major taxa (mean relative abundance, %)	Ref.
**Fallopian tube**						
Pelzer et al. (2018)	8	Australia	Fallopian tube dissection	454 pyrosequencing (V5-V8)	*Staphylococcus*	[15]
					*Escherichia*	
					*Pseudomonas*	
Zhou et al. (2019)	25	China	Fallopian tube fimbria tissue	Illumina Miseq (V3-V4)	*Proteobacteria*	[16]
					*Firmicutes*	
					*Bacteroidetes*	
					*Actinobacteria*	
					*Chlorofexi*	
					*Acidobacteria*	
					*Fusobacteria*	
**Endometrium**						
Fang et al. (2016)	10	China	Endometrial swabs	Illumina Miseq (V4)	*Enterobacter* (33.41%)	[17]
					*Pseudomonas* (23.56%)	
					*Lactobacillus* (6.23%)	
					*Desulfosporosinus* (4.33%)	
					*Ralstonia* (4.26%)	
					*Gardnerella* (3.55%)	
					*Cupriavidus* (0.92%)	
					*Prevotella* (0.83%)	
					*Thalassospira* (0.79%)	
					*Sphingomonas* (0.77%)	
					*Vibrio* (0.74%)	
					*Streptococcus* (0.59%)	
					*Atopobium* (0.58%)	
					*Bifidobacterium* (0.58%)	
					*Klebsiella* (0.53%)	
					*Megasphaera* (0.52%)	
					*Pelomonas* (0.51%)	
					*Alteromonas* (0.45%)	
					*Marinobacter* (0.24%)	
					*Erythrobacter* (0.22%)	
					*Veillonella* (0.21%)	
					*Muricauda* (0.19%)	
					*Methylobacterium* (0.19%)	
					*Escherichia* (0.18%)	
					*Bacillus* (0.17%)	
					*Mobiluncus* (0.16%)	
					*Singulisphaera* (0.16%)	
					*Tolumonas* (0.15%)	
					*Dialister* (0.14%)	
					*Thiothrix* (0.14%)	
					*Sneathia* (0.13%)	
					*Halomonas* (0.11%)	
					*Gemmata* (0.11%)	
					*Acinetobacter* (0.10%)	
					*Aquabacterium* (0.10%)	
					*Simkania* (0.10%)	
Moreno et al. (2016)	44	Spain	Endometrial fluid (aspirate)	454 pyrosequencing (V3-V5)	*Lactobacillus* (71.70%)	[18]
					*Gardnerella* (12.60%)	
					*Bifidobacterium* (3.70%)	
					*Streptococcus* (3.20%)	
					*Prevotella* (0.87%)	
Kyono et al. (2018)	15	Japan	Endometrial fluid (aspirate)	Illumina MiSeq (V4)	*Lactobacillus* (99.50%)	[19]
**Cervix**						
Filardo et al. (2017)	7	Italy	Endo-cervical swab	Illumina MiSeq (V3-V4)	*Lactobacillus* (96.2%)	[20]
					*Gardnerella*	
					*Atopobium*	
					*Bifidobacterium*	
Di Pietro et al. (2018)	7	Italy	Endo-cervical swab	Illumina MiSeq (V3-V4)	*Lactobacillus* (96%)	[21]
					*Gardnerella*	
					*Atopobium*	
					*Bifidobacterium*	
Graspeuntner et al. (2018)	89	Germany	Cervical swab	Illumina MiSeq (V3-V4)	*Lactobacillus* (78.34%)	[22]
					*Gardnerella* (5.43%)	
					*Prevotella* (3.02%)	
					*Bifidobacterium* (2.45%)	
					*Streptococcus* (1.75%)	
					*Enterobacteriaceae*, unclassified (1.70%)	
					*Atopobium* (1.61%)	
					*Aerococcus* (0.72%)	
					*Dialister* (0.59%)	
					*Sneathia* (0.56%)	
					*Veillonella* (0.56%)	
					*Porphyromonas* (0.26%)	
					*Clostridiales*, unclassified (0.12%)	
Ata et al. (2019)	14	Turkey	Endocervical swab	Illumina MiSeq (V3-V4)	*Lactobacillus*	[23]
					*Gardnerella*	
					*Prevotella*	
					*Atopobium*	
					*Dialister*	
Chorna et al. (2020)	8	Puerto Rico	Cervical swab	Not specified	*Lactobacillus*	[24]
					*Sneathia*	
					*Prevotella*	
					*Gardnerella*	
					*Atopobium*	
					*Shuttleworthia*	
Tu et al. (2020)	50	China	Cervical canal swabs	Illumina MiSeq (V3-V4)	*Lactobacillus*	[25]
					*Gardnerella*	
					*Atopobium*	
					*Sneathia*	
					*Ureaplasma*	
Wei et al. (2020)	14	China	Cervical mucus	Ion Torrent PGM (V4-V5)	*Lactobacillus* (64.3%)	[26]
Qingqing et al. (2021)	5	China	Not specified	Ion S5 ™ XL (V4)	*Lactobacillus* (90.01%)	[27]
**Vagina**						
Fang et al. (2016)	10	China	Vaginal swab	Illumina Miseq (V4)	*Lactobacillus* (60.93%)	[17]
					*Gardnerella* (15.30%)	
					*Prevotella* (6.28%)	
					*Enterobacter* (3.27%)	
					*Pseudomonas* (2.44%)	
					*Atopobium* (1.81%)	
					*Streptococcus* (1.32%)	
					*Megasphaera* (1.20%)	
					*Bifidobacterium* (0.97%)	
					*Sneathia* (0.55%)	
					*Desulfosporosinus* (0.40%)	
					*Dialister* (0.38%)	
					*Veillonella* (0.34%)	
					*Mobiluncus* (0.32%)	
					*Azorhizophilus* (0.18%)	
					*Ralstonia* (0.12%)	
Hong et al. (2016)	30	Korea	Vaginal swab	454 pyrosequencing (V3-V5)	*Lactobacillus* (83.41%)	[28]
					*Streptococcus* (4.90%)	
					*Diaphorobacter* (2.50%)	
					*Enterobacteriaceae* (1.97%)	
					*Cupriavidus* (1.36%)	
					*Prevotella* (0.80%)	
					*Cloacibacterium* (0.43%)	
					*Veillonella* (0.34%)	
					*Chlamydia* (0.22%)	
					*Comamonas* (0.20%)	
					*Novosphingobium* (0.18%)	
					*Staphylococcus* (0.16%)	
					*Haemophilus* (0.14%)	
					*Gemella* (0.13%)	
					*Pseudomonas* (0.11%)	
					*Acinetobacter* (0.10%)	
Moreno et al. (2016)	26	Spain	Vaginal aspirates	454 pyrosequencing (V3-V5)	*Lactobacillus*	[18]
					*Gardnerella*	
					*Atopobium*	
					*Prevotella*	
					*Sneathia*	
Campisciano et al. (2017)	30	Italy	Cervico-vaginal fluid	Ion Torrent PGM (V1-V3)	*Firmicutes; Bacilli* (97%)	[29]
					*Proteobacteria; Gammaproteobacteria* (1%)	
					*Bacteria; Actinobacteria*	
					*Bacteria; Tenericutes*	
Bradley et al. (2018)	47	Sweden	Cervicovaginal swab	454 pyrosequencing (V3-V4)	*Lactobacillus* (67.6%)	[30]
					*Gardnerella* (17.4%)	
					*Atopobium* (5.6%)	
					*Megasphaera* (3.3%)	
					*Prevotella* (2.2%)	
					*Sneathia*	
					*Coriobacteriaceae*	
					*Veillonella*	
					*Clostridium*	
Brotman et al. (2018)	30	United States	Vaginal swab	454 pyrosequencing (V1-V2)	*Lactobacillus* (83%)	[31]
Chen et al. (2018)	19	Taiwan	Vaginal swab	Illumina MiSeq (V4)	*Lactobacillus* (74%)	[32]
					*Bifidobacterium* (7%)	
					*Gardnerella*	
					*Prevotella*	
					*Atopobium*	
					*Escherichia*	
					*Dialister*	
Kyono et al. (2018)	15	Japan	Vaginal discharge (swab)	Illumina MiSeq (V4)	*Lactobacillus* (99.80%)	[19]
Matsumoto et al. (2018)	22	Japan	Vaginal swab	Illumina MiSeq (V3-V4)	*Lactobacillus*	[33]
					*Bifidobacterium*	
					*Gardnerella*	
					*Bacteroides*	
					*Escherichia*	
					*Enterococcus*	
					*Clostridium*	
Ata et al. (2019)	14	Turkey	Vaginal swab	Illumina MiSeq (V3-V4)	*Lactobacillus*	[23]
					*Gardnerella*	
					*Prevotella*	
					*Gemella*	
					*Megasphaera*	
					*Atopobium*	
					*Ureaplasma*	
					*Dialister*	
					*Sneathia*	
Ceccarani et al. (2019)	21	Italy	Vaginal swab	Illumina MiSeq (V3-V4)	*Lactobacillus* (79.16%)	[34]
					*Gardnerella* (2.72%)	
					Uncl. *Clostridiales* (1.66%)	
					*Faecalibacterium* (1.49%)	
					*Ruminococcaceae* (other) (1.35%)	
					*Prevotella* (1.16%)	
					*Roseburia* (1.09%)	
					Uncl. *Ruminococcaceae* (1.08%)	
					*Bacteroides* (0.69%)	
					*Oscillospira* (0.65%)	
					*Coprococcus* (0.61%)	
					*Ruminococcus* (0.54%)	
					*Anaerococcus* (0.48%)	
					*Streptococcus* (0.40%)	
					Uncl. *Lachnospiraceae* (0.40%)	
					*Dialister* (0.37%)	
					*Blautia* (0.35%)	
					*Peptoniphilus* (0.35%)	
					*Akkermansia* (0.30%)	
					*Porphyromonas* (0.25%)	
					*Ureaplasma* (0.25%)	
					*Bifidobacterium* (0.20%)	
					*Parvimonas* (0.20%)	
					*Sneathia* (0.18%)	
					*Atopobium* (0.17%)	
					*Clostridium* (0.16%)	
					*Escherichia* (0.13%)	
					Uncl. *Coriobacteriaceae* (0.10%)	
Hong et al. (2019)	37	China	Vaginal swab	Illumina HiSeq (V3-V4)	*Lactobacillus*	[35]
					*Gardnerella*	
					*Atopobium*	
					*Prevotella*	
					*Streptococcus*	
					*Sneathia*	
Lin et al. (2019)	16	China	Vaginal secretion	Illumina MiSeq (V3-V4)	*Lactobacillus* (43.88%)	[36]
					*Bifidobacteriaceae* (16.54%)	
					*Streptococcus* (9.82%)	
					*Coriobacteriaceae* (7.22%)	
Liu et al. (2019)	30	China	Vaginal swab	Illumina HiSeq (V4)	*Lactobacillus* (>97%)	[37]
Xu et al. (2019)	32	China	Vaginal swab	Illumina MiSeq (V3-V4)	*Lactobacillus* (83.80%)	[38]
					*Gardnerella* (3.19%)	
					*Sneathia* (2.26%)	
Zhou et al. (2019)	42	China	Vaginal swab	Illumina MiSeq (V3-V4)	*Lactobacillus* (86.59%)	[39]
					*Gardnerella* (3.26%)	
					*Pseudomonas* (3.23%)	
					*Prevotella* (2.01%)	
					*Atopobium* (1.70%)	
					*Dialister* (0.24%)	
					*Anaerococcus* (0.23%)	
					*Aerococcus* (0.18%)	
					*Stenotrophomonas* (0.17%)	
					*Megasphaera* (0.16%)	
					*Bacteroides* (0.13%)	
Chen et al. (2020)	68	China	Vaginal swab	Illumina MiSeq (V3-V4)	*Lactobacillus* (64.93%)	[40]
					*Gardnerella*	
					*Prevotella* (5.91%)	
					*Atopobium* (3.12%)	
					*Sneathia* (2.39%)	
					*Anaerococcus* (1.22%)	
					*Streptococcus* (1.03%)	
					*Megasphaera* (1.01%)	
					*Bacillus* (0.34%)	
Chorna et al. (2020)	8	Puerto Rico	Vaginal swab	Not specified	*Lactobacillus*	[24]
					*Shuttleworthia*	
					*Gardnerella*	
					*Atopobium*	
					*Prevotella*	
					*Megasphaera*	
					*Sneathia*	
Tu et al. (2020)	50	China	Vaginal swab	Illumina MiSeq (V3-V4)	*Lactobacillus*	[25]
					*Gardnerella*	
					*Atopobium*	
Wang et al. (2020)	160	China	Vaginal swab	Illumina HiSeq (V4)	*Lactobacillus* (95.90%)	[41]
					*Gardnerella*	
					*Pseudomonas*	
					*Streptococcus*	
					*Aerococcus*	
					*Atopobium*	
					*Prevotella*	
Wang et al. (2020)	29	China	Vaginal swab	Illumina MiSeq (V3-V4)	*Lactobacillus*	[42]
					*Gardnerella*	
					*Bacteroides*	
					*Prevotella*	
					*Atopobium*	
Xie et al. (2020)	27	China	Vaginal swab	Illumina MiSeq (V4)	*Lactobacillus*	[43]
					*Acinetobacter*	
					*Megasphaera*	
					*Pseudomonas*	
					*Ochrobactrum*	
					*Sneathia*	
Zhao et al. (2020)	92	China	Vaginal swab	Illumina HiSeq (V1-V2)	*Lactobacillus*	[44]
					*Bifidobacterium*	
					*Prevotella*	
					*Atopobium*	
					*Bacteroides*	
					*Streptococcus*	
					*Clostridium*	
Sirichoat et al. (2021)	51	Thailand	Vaginal swab	Ion Torrent PGM (V2, V3, V4, V6-7, V8, V9)	*Lactobacillus* (78%)	[45]
					*Gardnerella* (14%)	
					*Atopobium* (2%)	
					*Pseudomonas* (2%)	

## Diversity of the Female Genital Tract Microbiome in Women With Health Conditions Associated With Ectopic Pregnancy

**FIGURE 1:**
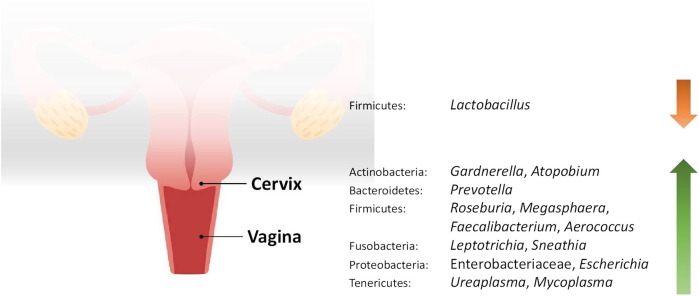
Changes in the relative abundance of the vaginal and cervical microbiome in reproductive conditions compared to the healthy controls.

**FIGURE 2:**
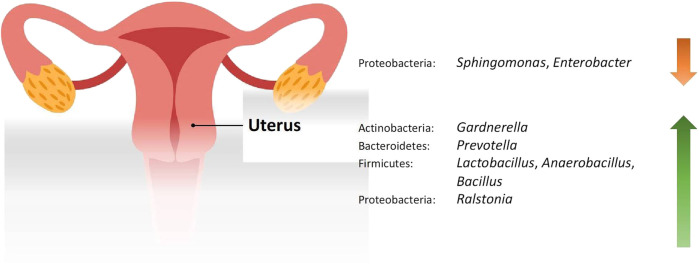
Changes in the relative abundance of the uterine microbiome in reproductive conditions compared to the healthy controls.

Studies included for women with health conditions were cross-sectional except for an observational prospective study investigating the vaginal microbiome in women with failed intrauterine insemination [46]. Meanwhile, the sample size of the studies also varied with a range of 1–118. Not all studies provided numerical values of relative abundance and these were ranked according to descending abundance. For standardisation, the lowest taxonomic rank observed in our review is the genus level while taxa with a relative abundance of less than 0.1% were not tabulated. Table 2 summarises the relative abundance of vaginal microbiome in various reproductive conditions while Table 3 outlines the changes in relative abundance in comparison with healthy groups. Table 4 summarises the cervical microbiome’s relative abundance in reproductive conditions while Table 5 compares the relative abundance with healthy groups. Table 6 highlights the relative abundance of uterine microbiome in reproductive conditions while Table 7 shows the comparison of uterine microbiome relative abundance in disease state with healthy controls. Overall, there was a decrease in the relative abundance of the genus *Lactobacillus* in the disease state and an increase in various other genera in the vaginal and cervical microbiome (Figure 1). Meanwhile looking at the uterine microbiome, various sampling methods were used, with inconsistent findings across studies. However, in general, there was a decrease in the phylum Proteobacteria and an increase in the other taxa (Figure 2).

**TABLE 2:** Relative abundance of the vaginal microbiome in various reproductive conditions.

Reproductive condition	Author (Year)	Genital microbiome relative abundance (%)								
		Actinobacteria	Bacteroidetes	Candidatus Saccharibacteria	Cyanobacteria	Firmicutes	Fusobacteria	Proteobacteria	Tenericutes	Verrucomicrobia
Tubal pregnancy	Ruan (2021) [47]	*Gardnerella* 12	*Prevotella* 6			*Lactobacillus* 62	*Sneathia* 3			
		*Atopobium* 4				*Megasphaera* 2				
Chronic endometritis	Lozano (2021) [48]		*Prevotella* 0.98			*Lactobacillus* 87.44		*Escherichia* 0.17		
						*Streptococcus* 9.44				
						*Dialister* 0.68				
						*Veillonella* 0.68				
*Chlamydia trachomatis*	Ceccarani (2019) [34]	*Gardnerella* 3.65	*Prevotella* 1.6			*Lactobacillus* 67.45	*Sneathia* 0.41	*Escherichia* 0.33		*Akkermansia* 0.39
		*Atopobium* 1	*Bacteroides* 0.91			*Roseburia* 4.42		*Haemophilus* 0.1		
		*Bifidobacterium* 0.46				*Megasphaera* 2.97				
		Coriobacteriaceae, unclassified^a^ 0.19				*Faecalibacterium* 2.31				
						Ruminococcaceae^a^ 1.95				
						Clostridiales, unclassified^a^ 1.56				
						Ruminococcaceae, unclassified^a^ 1.03				
						*Blautia* 0.91				
						*Coprococcus* 0.66				
						*Clostridium* 0.65				
						Lachnospiraceae, unclassified^a^ 0.59				
						*Ruminococcus* 0.58				
						*Dialister* 0.56				
						*Oscillospira* 0.56				
						*Shuttleworthia* 0.54				
						*Streptococcus* 0.49				
						*Aerococcus* 0.24				
						*Peptoniphilus* 0.15				
Vulvovaginal candidiasis	Ceccarani (2019) [34]	*Gardnerella* 7.68	*Prevotella* 3.76			*Lactobacillus* 56.69	*Sneathia* 0.53	*Haemophilus* 1.42	*Ureaplasma* 0.41	*Akkermansia* 0.35
		*Atopobium* 1.94	*Bacteroides* 0.81			*Roseburia* 3.51		*Escherichia* 0.4		
		*Bifidobacterium* 1.28				*Faecalibacterium* 2.14				
		*Alloscardovia* 0.57				Ruminococcaceae^a^ 1.86				
		Coriobacteriaceae,				*Aerococcus* 1.5				
		*unclassified* ^a^ *0.24*				Clostridiales, unclassified^a^ 1.44				
						*Megasphaera* 1.04				
						*Streptococcus* 1.04				
						Ruminococcaceae, unclassified^a^ 1.02				
						*Dialister* 0.78				
						*Blautia* 0.77				
						*Coprococcus* 0.63				
						*Ruminococcus* 0.59				
						Lachnospiraceae, unclassified^a^ 0.54				
						*Oscillospira* 0.53				
						Gemellaceae, unclassified^a^ 0.49				
						*Veillonella* 0.46				
						*Anaerococcus* 0.46				
						*Finegoldia* 0.45				
						*Gemella* 0.37				
						*Clostridium* 0.32				
						*Shuttleworthia* 0.31				
						*Parvimonas* 0.17				
						*Peptoniphilus* 0.13				
LR-HPV Infection	Zhou (2019) [39]	*Gardnerella* 10.83	*Prevotella* 4.17			*Lactobacillus* 49.95	*Sneathia* 5.69	*Pseudomonas* 1.57		
		*Atopobium* 4.62	*Bacteroides* 1.89			*Saccharofermentans* 1.33	*Fusobacterium* 0.66	*Hydrogenophilus* 0.55		
		*Bifidobacterium* 2.43				*Megasphaera* 1.12		*Burkholderia* 0.48		
		*Corynebacterium* 1.33				*Peptostreptococcus* 0.62		*Escherichia/Shigella* 0.30		
						*Stenotrophomonas* 0.57				
						*Dialister* 0.42				
						*Aerococcus* 0.27				
						*Anaerococcus* 0.25				
Bacterial vaginosis	Ceccarani (2019) [34]	*Gardnerella* 11.44	*Prevotella* 9.15	Rs-045, unclassified^a^ 0.43		*Lactobacillus* 18.8	*Sneathia* 7.76	*Escherichia* 0.23		*Akkermansia* 0.32
		*Atopobium* 4.92	*Bacteroides* 0.86			*Megasphaera* 8.64				
		Coriobacteriaceae, unclassified^a^ 0.89	*Porphyromonas* 0.73			*Shuttleworthia* 7.48				
		*Mobiluncus* 0.49				*Roseburia* 3.51				
		*Bifidobacterium* 0.33				*Clostridium* 2.14				
						*Faecalibacterium* 2.09				
						*Aerococcus* 2.06				
						*Dialister* 2.02				
						Ruminococcaceae^a^ 1.81				
						Clostridiales, unclassified^a^ 1.58				
						*Parvimonas* 1.39				
						*Peptoniphilus* 1.09				
						Ruminococcaceae, unclassified^a^ 1.03				
						*Blautia* 0.74				
						*Peptostreptococcus* 0.63				
						*Coprococcus* 0.59				
						*Oscillospira* 0.58				
						*Ruminococcus* 0.56				
						*Streptococcus* 0.54				
						Lachnospiraceae, unclassified^a^ 0.52				
						*Anaerococcus* 0.39				
						*Gemella* 0.27				
						*Finegoldia* 0.16				
Bacterial vaginosis	Hong (2016) [28]	*Atopobium* 4.46	*Prevotella* 27.80			*Lactobacillus* 38.98	*Sneathia* 7.48	*Diaphorobacter* 1.67	*Mycoplasma* 0.35	
		*Gardnerella* 1.36	*Porphyromonas* 1.29			*Aerococcus* 5.62	*Fusobacterium* 0.30	*Cupriavidus* 1.03		
		*Mobiluncus* 0.69				*Megasphaera* 1.72				
		Coriobacteriaceae, unclassified^a^ 0.38				*Dialister* 1.05				
						*Saccharofermentans* 0.94				
						*Peptoniphilus* 0.69				
						*Anaerococcus* 0.55				
						*Moryella* 0.35				
Vaginosis	Campisciano (2017) [29]	Actinobacteria^a^ 16	Bacteroidia^a^ 5			Bacilli^a^ 71	Fusobacteria^a^ 1	Gammaproteobacteria^a^ 4	Tenericutes^a^ 1	
						Clostridia^a^ 1				
Aerobic vaginitis	Wang (2019) [41]	*Gardnerella*	*Prevotella*			*Lactobacillus* 41.6	*Sneathia*	*Klebsiella* 0.5	*Ureaplasma* 0.3	
		*Atopobium*				*Streptococcus*		*Escherichia*	*Mycoplasma* 0.12	
		*Bifidobacterium*				*Aerococcus*				
		*Alloscardovia*				*Anaerococcus*				
						*Eubacterium*				
						*Veillonella*				
						*Megasphaera*				
						*Dialister*				
Empty-sac miscarriage	Liu (2021) [49]		*Bacteroides*			*Lactobacillus*		*Halomonas*		
Missed miscarriage	Liu (2021) [49]		*Bacteroides*		Cyanobacteria^a^	*Lactobacillus*	*Fusobacterium*	*Halomonas*		
						Lachnospiraceae^a^		*Escherichia/Shigella*		
						*Bacillus*		*Succinivibrio*		
						*Staphylococcus*		*Burkhoderia*		
								*Acetobacter*		
Embryonic miscarriage	Xu (2020) [50]	*Bifidobacterium*	*Bacteroides*			*Lactobacillus*		*Escherichia-Shigella*		
		*Gardnerella*	*Parabacteroides*			*Faecalibacterium*				
			*Alistipes*			Lachnospiraceae^a^				
						*Roseburia*				
ART failure	Bernabeu (2019) [51]	*Gardnerella*				*Lactobacillus*			*Ureaplasma*	
						*Streptococcus*				
						*Clostridium*				
IUI failure	Amato (2020) [46]	Bifidobacteriaceae^a^ 12				Lactobacillaceae^a^ 83				
IVF failure	Kong (2020) [52]	*Gardnerella* 7.24	*Prevotella* 3.02			*Lactobacillus* 63.09	*Sneathia* 3.75	Proteobacteria^a^ 8.01		
		*Atopobium* 4.14				*Streptococcus*				
						*Megasphaera*				
						*Aerococcus*				
Infertility	Riganelli (2020) [53]	*Bifidobacterium*	*Prevotella*			*Lactobacillus*		*Escherichia*		
		*Gardnerella*				*Streptococcus*				
		*Atopobium*				*Shuttleworthia*				
	Zhao (2020) [44]	*Bifidobacterium*	*Prevotella*			*Lactobacillus*				
		*Atopobium*				*Aerococcus*				
Idiopathic infertility	Campisciano (2017) [29]	Actinobacteria^a^ 8	Bacteroidia^a^ 1			Bacilli^a^ 84		Gammaproteobacteria^a^ 3	Tenericutes^a^ 2	
						Clostridia^a^ 1				
Diagnosed infertility	Campisciano (2017) [29]	Actinobacteria^a^ 5				Bacilli^a^ 71		Gammaproteobacteria^a^ 23		
						Clostridia^a^ 1				
Deep endometriosis	Hernandes (2020) [54]	*Gardnerella*	*Prevotella*			*Lactobacillus*		*Pseudomonas*	*Ureaplasma*	
		*Corynebacterium*				*Streptococcus*		*Alishewanella*		
						*Enterococcus*				
						*Anaerococcus*				
PCOS	Hong (2021) [55]	*Gardnerella* 10.4	*Prevotella* 7.94			*Lactobacillus* 58.52	*Sneathia* 1.57	*Mycoplasma* 1.25		
		*Atopobium* 4.36				*Streptococcus* 2.76				
		*Bifidobacterium* 1.55				*Megasphaera* 1.54				
	Tu (2020) [25]	*Gardnerella*	*Prevotella*			*Lactobacillus*	*Sneathia*	*Escherichia-Shigella*	*Ureaplasma*	
		*Atopobium*	*Porphyromonas*			*Streptococcus*	*Fusobacterium*	*Campylobacter*	*Mycoplasma*	
		*Bifidobacterium*				*Aerococcus*		*Acinetobacter*		
		*Corynebacterium*				*Dialister*				
		*Lawsonella*				*Peptoniphilus*				
						*Finegoldia*				
						*Anaerococcus*				
						*Veillonella*				
						*Megasphaera*				
						*Peptostreptococcus*				
						*Varibaculum*				
						*Staphylococcus*				
						*Ezakiella*				
Intrauterine adhesion	Liu (2019) [37]	Actinobacteria^a^ 24.37	Bacteroidetes^a^ 8.64			Firmicutes^a^ 61.84		Proteobacteria^a^ 2.74		

^a^ Unknown genera.

**TABLE 3:** Comparison of the vaginal microbiome relative abundance in disease state with healthy controls.

Reproductive condition	Author (Year)	Genital microbiome relative abundance (%)							
		Actinobacteria	Bacteroidetes	Candidatus Saccharibacteria	Firmicutes	Fusobacteria	Proteobacteria	Tenericutes	Verrucomicrobia
Tubal pregnancy	Ruan (2021) [47]	*Gardnerella* ↑	*Prevotella ↑*		*Lactobacillus*	*Sneathia*			
		*Atopobium*			*Megasphaera*	Leptotrichiaceae^a^ ↑			
					Clostridia^a^ ↑				
Chronic endometritis	Lozano (2021) [48]		*Prevotella*		*Lactobacillus ↓*		*Escherichia*		
					*Streptococcus ↑*				
					*Dialister*				
					*Veillonella*				
*Chlamydia trachomatis*	Ceccarani (2019) [34]	*Gardnerella*	*Prevotella*		*Lactobacillus*	*Sneathia ↑*	*Escherichia* ↑		*Akkermansia*
		*Atopobium ↑*	*Bacteroides ↑*		*Roseburia ↑*		*Haemophilus*		
		*Bifidobacterium ↑*			*Megasphaera ↑*				
		Coriobacteriaceae, unclassified^a^ ↑			*Faecalibacterium ↑*				
					Ruminococcaceae^a^ ↑				
					Clostridiales, unclassified^a^				
					Ruminococcaceae, unclassified^a^				
					*Blautia ↑*				
					*Coprococcus ↑*				
					*Clostridium ↑*				
					Lachnospiraceae, unclassified^a^ ↑				
					*Ruminococcus*				
					*Dialister ↑*				
					*Oscillospira*				
					*Shuttleworthia ↑*				
					*Streptococcus ↑*				
					*Aerococcus ↑*				
					*Peptoniphilus*				
Vulvovaginal candidiasis	Ceccarani (2019) [34]	*Gardnerella ↑*	*Prevotella ↑*		*Lactobacillus ↓*	*Sneathia*	*Haemophilus*	*Ureaplasma*	*Akkermansia*
		*Atopobium ↑*	*Bacteroides* ↑		*Roseburia*		*Escherichia ↑*		
		*Bifidobacterium ↑*			*Faecalibacterium ↑*				
		*Alloscardovia ↑*			Ruminococcaceae^a^ ↑				
		Coriobacteriaceae, unclassified^a^ ↑			*Aerococcus*				
					Clostridiales, unclassified^a^				
					*Megasphaera ↑*				
					*Streptococcus ↑*				
					Ruminococcaceae, unclassified^a^				
					*Dialister ↑*				
					*Blautia ↑*				
					*Coprococcus ↑*				
					*Ruminococcus*				
					Lachnospiraceae, unclassified^a^ ↑				
					*Oscillospira*				
					Gemellaceae, unclassified^a^				
					*Veillonella ↑*				
					*Anaerococcus*				
					*Finegoldia ↑*				
					*Gemella*				
					*Clostridium*				
					*Shuttleworthia ↑*				
					*Parvimonas*				
					*Peptoniphilus*						
LR-HPV Infection	Zhou (2019) [39]	*Gardnerella ↑*	*Prevotella*		*Lactobacillus ↓*	*Sneathia ↑*	*Pseudomonas*		
		*Atopobium*	*Bacteroides*		*Saccharofermentans*	*Fusobacterium*	*Hydrogenophilus*		
		*Bifidobacterium*			*Megasphaera*		*Burkholderia*		
		*Corynebacterium*			*Peptostreptococcus*		*Escherichia/Shigella*		
					*Stenotrophomonas*				
					*Dialister*				
					*Aerococcus*				
					*Anaerococcus*				
Bacterial vaginosis	Ceccarani (2019) [34]	*Gardnerella ↑*	*Prevotella ↑*	Rs-045, unclassified^a^	*Lactobacillus ↓*	*Sneathia ↑*	*Escherichia*		*Akkermansia*
		*Atopobium ↑*	*Bacteroides ↑*		*Megasphaera ↑*				
		Coriobacteriaceae, unclassified^a^ ↑	*Porphyromonas*		*Shuttleworthia ↑*				
		*Mobiluncus ↑*			*Roseburia*				
		*Bifidobacterium*			*Clostridium*				
					*Faecalibacterium*				
					*Aerococcus ↑*				
					*Dialister ↑*				
					Ruminococcaceae^a^				
					Clostridiales, unclassified^a^				
					*Parvimonas*				
					*Peptoniphilus*				
					Ruminococcaceae, unclassified^a^				
					*Blautia*				
					*Peptostreptococcus ↑*				
					*Coprococcus*				
					*Oscillospira*				
					*Ruminococcus*				
					*Streptococcus*				
					Lachnospiraceae, unclassified^a^ ↑				
					*Anaerococcus*				
					*Gemella ↑*				
					*Finegoldia*				
Vaginosis	Campisciano (2017) [29]	Actinobacteria^a^	Bacteroidia^a^		Bacilli^a^ ↓	Fusobacteria^a^	Gammaproteobacteria^a^ ↑	Tenericutes^a^ ↑	
					Clostridia^a^				
Aerobic vaginitis	Wang (2019) [41]	*Gardnerella* ↑	*Prevotella* ↑		*Lactobacillus* ↓	*Sneathia*	*Klebsiella*	*Ureaplasma*	
		*Atopobium* ↑	Bacteroidetes^a^		*Streptococcus* ↑		*Escherichia*	*Mycoplasma*	
		*Bifidobacterium*			*Aerococcus* ↑				
		*Alloscardovia*			*Anaerococcus*				
		Actinobacteria^a^			*Eubacterium*				
					*Veillonella*				
					*Megasphaera*				
					*Dialister*				
Embryonic miscarriage	Xu (2020) [50]	*Bifidobacterium*	*Bacteroides*		*Lactobacillus*	Fusobacteria^a^ ↓	*Escherichia-Shigella*		
		*Gardnerella*	*Parabacteroides*		*Faecalibacterium*				
			*Alistipes*		Lachnospiraceae^a^ ↑				
					*Roseburia ↑*				
IUI failure	Amato (2020) [46]	Bifidobacteriaceae^a^ ↑			Lactobacillaceae^a^ ↓				
Idiopathic infertility	Campisciano (2017) [29]	Actinobacteria^a^ ↑	Bacteroidia^a^		Bacilli^a^		Gammaproteobacteria^a^	Tenericutes^a^	
					Clostridia^a^				
PCOS	Tu (2020) [25]	*Gardnerella*	*Prevotella* ↑		*Lactobacillus*	*Sneathia*	*Escherichia-Shigella*	*Ureaplasma*	
		*Atopobium*	*Porphyromonas* ↑		*Streptococcus*	*Fusobacterium*	*Campylobacter*	*Mycoplasma* ↑	
		*Bifidobacterium*			*Aerococcus*		*Acinetobacter*		
		*Corynebacterium*			*Dialister*				
		*Lawsonella*			*Peptoniphilus* ↑				
					*Finegoldia*				
					*Anaerococcus*				
					*Veillonella*				
					*Megasphaera*				
					*Peptostreptococcus*				
					*Varibaculum*				
					*Staphylococcus*				
					*Ezakiella*				
Intrauterine adhesion	Liu (2019) [37]	Actinobacteria^a^	Bacteroidetes^a^ ↑		Firmicutes^a^		Proteobacteria^a^		

^a^ Unknown genera.

**TABLE 4:** Relative abundance of the cervical microbiome in various reproductive conditions.

Reproductive condition	Author (Year)	Genital microbiome relative abundance (%)							
		Actinobacteria	Bacteroidetes	Chlamydiae	Cyanobacteria	Firmicutes	Fusobacteria	Proteobacteria	Tenericutes
*Chlamydia trachomatis*	Di Pietro (2018) [21]	*Gardnerella* 14	*Prevotella* 6			*Lactobacillus* 50	*Leptotrichia* 21		
	Filardo (2019) [56]	*Gardnerella* 15.5	*Prevotella* 6.5			*Lactobacillus* 51.1			
						*Aerococcus* 1			
Asymptomatic *Chlamydia trachomatis*	Filardo (2017) [20]	*Gardnerella* 14.3	*Prevotella* 0.5	*Chlamydia*		*Lactobacillus* 60	*Leptotrichia* 10	*Escherichia*	*Ureaplasma*
		*Atopobium*				*Megasphera*	*Fusobacterium*		*Mycoplasma*
		10 *Bifidobacterium*				*Dialister*			
						*Streptococcus*			
						*Aerococcus*			
						*Parvimonas*			
HPV/CT	Di Pietro (2018) [21]	*Gardnerella* 19	Bacteroidetes^a^ *<*1			Firmicutes^a^ 63	Fusobacteria^a^ <1		
		*Atopobium* 4							
HPV	Di Pietro (2018) [21]	Actinobacteria^a^ 1.3	Bacteroidetes^a^ *<*1			Firmicutes^a^ 98	Fusobacteria^a^ <1	Proteobacteria^a^ <1	*Tenericutes* ^a^ *<*1
HPV infection - LSIL	Kwasniewski (2018) [57]	Actinobacteria^a^ 1				Bacilli^a^ 84		Gammproteobacteria^a^ 8.2	
						Clostridia^a^ 0.1			
HPV infection—HSIL	Kwasniewski (2018) [57]	Actinobacteria^a^ 8.1			Nostocophycideae^a^ 0.15	Bacilli^a^ 27.69		Gammproteobacteria^a^ 61.48	
						Clostridia^a^ 0.2		Alphaproteobacteria^a^ 0.41	
							
Infectious infertility	Graspeuntner (2018) [22]	*Gardnerella* 10.08	*Prevotella* 7.37			*Lactobacillus* 57.74	*Sneathia* 2.58	Enterobacteriaceae, unclassified^a^ 0.27	*Mycoplasma* 1.71
		*Atopobium* 2.18	*Porphyromonas* 0.27			*Streptococcus* 5.5			
		*Corynebacterium* 1.28				Lachnospiraceae^a^ 1.69			
		*Bifidobacterium* 0.18				*Veillonella* 1.63			
						*Dialister* 1.25			
						*Aerococcus* 0.48			
Non-infectious infertility	Graspeuntner (2018) [22]	*Gardnerella* 5.61	*Prevotella* 3.93			*Lactobacillus* 69.01	*Sneathia* 0.5	Enterobacteriaceae, unclassified^a^ 0.98	*Mycoplasma* 0.02
		*Atopobium* 2.72				Lachnospiraceae^a^ 6.59			
		*Bifidobacterium* 0.21				*Aerococcus* 1.49			
						*Dialister* 0.94			
						*Veillonella* 0.76			
						*Streptococcus* 0.65			
						Clostridiales, unclassified^a^ 0.37			
Stage 3 endometriosis	Cregger (2017) [11]		*Barnesiella* 19.75			*Clostridium XIVa* 2.25	*Sneathia* 0.25	*Achromobacter* 0.75	
			*Bacteroides* 1.75			*Staphylococcus* 1.5			
			*Tannerella* 1.75			*Coprococcus* 1.25			
			*Parabacteroides* 1.25			*Propionibacterium* 1.25			
			*Alkalitalea* 0.75			*Allobaculum* 1			
						*Butyricicoccus* 1			
						*Acetivibrio* 0.75			
						*Anaerotruncus* 0.75			
						*Ruminococcus* 0.75			
						*Turicibacter* 0.75			
						*Coprobacillus* 0.5			
						*Lactobacillus* 0.5			
						*Clostridium XIVb* 0.25			
						*Flavonifractor* 0.25			
Endometriosis—other stages	Cregger (2017) [11]		*Barnesiella* 0.93						
Stage 3–4 endometriosis	Ata (2019) [23]	2 *Gardnerella*	3 *Prevotella*			*Lactobacillus*			
		5 *Atopobium*				*Dialister*			
						*Streptococcus*			
PCOS	Tu (2020) [25]	*Gardnerella*	*Prevotella*			*Lactobacillus*	*Sneathia*	*Escherichia-Shigella*	*Ureaplasma*
		*Atopobium*	*Porphyromonas*			*Streptococcus*	*Fusobacterium*	*Campylobacter*	*Mycoplasma*
		*Bifidobacteriu*				*Finegoldia*		*Acinetobacter*	
		*Varibaculum*				*Peptoniphilus*		*Sutterella*	
		*Corynebacterium*				*Aerococcus*			
						*Dialister*			
						*Megasphaera*			
						*Anaerococcus*			
						*Veillonella*			
						*Peptostreptococcus*			
						*Staphylococcus*			

^a^ Unknown genera.

**TABLE 5:** Comparison of the cervical microbiome relative abundance in disease state with healthy controls.

Reproductive condition	Author (Year)	Genital microbiome relative abundance (%)						
		Actinobacteria	Bacteroidetes	Chlamydiae	Firmicutes	Fusobacteria	Proteobacteria	Tenericutes
*Chlamydia trachomatis*	Di Pietro (2018) [21]	*Gardnerella ↑*	*Prevotella ↑*		*Lactobacillus ↓*	*Leptotrichia*		
	Filardo (2019) [56]	*Gardnerella ↑*	*Prevotella ↑*		*Lactobacillus ↓*			
					*Aerococcus*			
Asymptomatic *Chlamydia trachomatis*	Filardo (2017) [20]	*Gardnerella* ↑	*Prevotella* 0.5 ↑	*Chlamydia*	*Lactobacillus* ↓	*Leptotrichia* ↑	*Escherichia*	*Ureaplasma*
		*Atopobium* ↑			*Megasphera*	*Fusobacterium*		*Mycoplasma*
		*Bifidobacterium*			*Dialister*			
					*Streptococcus*			
					*Aerococcus*			
					*Parvimonas*			
HPV/CT	Di Pietro (2018) [21]	*Gardnerella ↑*	Bacteroidetes^a^		Firmicutes^a^	Fusobacteria^a^		
		*Atopobium ↑*						
PCOS	Tu (2020) [25]	*Gardnerella* ↑	*Prevotella* ↑		*Lactobacillus* ↓	*Sneathia*	*Escherichia-Shigella*	*Ureaplasma*
		*Atopobium*	*Porphyromonas* ↑		*Streptococcus*	*Fusobacterium*	*Campylobacter*	*Mycoplasma*
		*Bifidobacterium*			*Finegoldia* ↑		*Acinetobacter*	
		*Varibaculum*			*Peptoniphilus* ↑		*Sutterella*	
		*Corynebacterium*			*Aerococcus* ↑			
					*Dialister* ↑			
					*Megasphaera*			
					*Anaerococcus* ↑			
					*Veillonella*			
					*Peptostreptococcus*			
					*Staphylococcus*			

^a^ Unknown genera.

**TABLE 6:** Relative abundance of the uterine microbiome relative abundance in various reproductive conditions.

Type of sample	Reproductive condition	Author (Year)	Genital microbiome abundance						
			Actinobacteria	Bacteroidetes	Cyanobacteria	Firmicutes	Proteobacteria	Tenericutes	Verrucomicrobia
Endometrial fluid	Infertility	Vladislavovna (2020) [58]	*Gardnerella* 2.51			*Lactobacillus* 34.37	*Ralstonia* 7.23		
						*Streptococcus* 2.7	*Methylobacterium* 2.92		
							*Comamonas* 2.87		
	Infertility (pipelle catheter)	Riganelli (2020) [53]	Actinobacteria^a^	Bacteroidete^a^	Cyanobacteria^a^	Firmicutes^a^	Proteobacteria^a^		Verrucomicrobia^a^
						
	Infertility without chronic endometritis	Liu (2019) [59]	*Gardnerella ∼*8	*Prevotella ∼*1		*Lactobacillus ∼*58	*Stenotrophomonas ∼*3		
			*Atopobium ∼*5			*Streptococcus ∼*3	*Escherichia-Shigella ∼*1		
			*Bifidobacterium ∼*3			*Staphylococcus ∼*1			
	Failure of implantation	Moreno (2016) [18]	*Gardnerella*			*Lactobacillus*			
			*Bifidobacterium*			*Streptococcus*			
						*Veillonella*			
						Clostridiales, unclassified^a^			
	Miscarriage in infertile women	Moreno (2016) [18]	*Gardnerella*			*Lactobacillus*			
			*Bifidobacterium*			*Faecalibacterium*			
						*Ruminococcus*			
						*Roseburia*			
						Lachnospiraceae^a^			
						*Blautia*			
Endometrial swab	Endometrial polyps	Fang (2016) [17]	*Gardnerella* 5.5	*Prevotella* 1.3		*Lactobacillus* 38.64	*Enterobacter* 8.34		
			*Bifidobacterium* 4.8			*Desulfosporosinus* 4.23	*Pseudomonas* 7.02		
						*Streptococcus* 2.6	*Alteromonas* 1.1		
							Enterobacteriaceae, unclassified^a^ 0.9		
							*Sphingomonas* 0.4		
	Endometrial polyps/Chronic endometritis	Fang (2016) [17]	*Gardnerella* 6.91	*Prevotella* 1.3		*Lactobacillus* 33.21	*Pseudomonas* 7.32		
			*Bifidobacterium* 1.4			*Desulfosporosinus* 5.41	*Enterobacter* 7.17		
						*Streptococcus* 1.1	*Alteromonas* 1.4		
							Enterobacteriaceae, unclassified^a^ 1		
							*Sphingomonas* 0.6		
	Chronic endometritis	Lozano (2021) [48]	*Gardnerella* 4.05			*Lactobacillus* 81.76	*Burkholderia* 3.38		
						*Anaerobacillus* 2.03	*Ralstonia* 2.7		
						*Dialister* 2.03	*Delftia* 1.35		
						*Streptococcus* 2.03			
	Endometriosis	Khan (2016) [60]				Lactobacillaceae^a^ 27	Moraxellaceae^a^ 15		
						Streptococcaceae^a^ 11	Enterobacteriaceae^a^ 1		
						Staphylococcaceae^a^ 5			
Endometrial tissue	Deep endometriosis	Hernandes (2020) [54]	*Corynebacterium*	*Prevotella*		*Lactobacillus*	*Alishewanella*	*Ureaplasma*	
			*Gardnerella*			*Enterococcus*	*Pseudomonas*		
						*Anaerococcus*			

^a^ Unknown genera.

**TABLE 7:** Comparison of the uterine microbiome relative abundance in disease state with healthy controls.

Type of sample	Reproductive condition	Author (Year)	Genital microbiome relative abundance (%)			
			Actinobacteria	Bacteroidetes	Firmicutes	Proteobacteria
Endometrial fluid	Miscarriage in infertile women	Moreno (2016) [18]	*Gardnerella* ↑		*Lactobacillus*	
			*Bifidobacterium*		*Faecalibacterium*	
					*Ruminococcus*	
					*Roseburia*	
					Lachnospiraceae^a^	
					*Blautia*	
Endometrial swab	Endometrial polyps	Fang (2016) [17]	*Gardnerella*	*Prevotella*	*Lactobacillus*	*Enterobacter*
			*Bifidobacterium*		*Desulfosporosinus*	*Pseudomonas*
					*Streptococcus*	*Alteromonas*
					Firmicutes^a^ ↑	Enterobacteriaceae, unclassified^a^
						*Sphingomonas*
						Proteobacteria^a^ ↓
	Endometrial polyps/Chronic endometritis	Fang (2016) [17]	*Gardnerella*	*Prevotella ↑*	*Lactobacillus*	*Pseudomonas*
			*Bifidobacterium*		*Desulfosporosinus*	*Enterobacter ↓*
					*Streptococcus*	*Alteromonas*
					Firmicutes^a^ ↑	Enterobacteriaceae, unclassified^a^
						*Sphingomonas* ↓
						Proteobacteria^a^ ↓
	Chronic endometritis	Lozano (2021) [48]	*Gardnerella*		*Lactobacillus* ↓	*Burkholderia*
					*Anaerobacillus*	*Ralstonia*
					*Dialister ↓*	*Delftia*
					*Streptococcus*	

^a^ Unknown genera.

## The Genital Tract Microbiome Among Healthy, Fertile Women Is Dominated by *Lactobacillus*

The microbiome of the female reproductive system is best understood when described from the lower to the upper genital tract. All studies in healthy women demonstrated that *Lactobacillus* dominates the vagina*.* This Gram-positive rod bacterium provides a major source of vaginal lactic acid by processing glycogen and its byproducts. Human α-amylase catabolises glycogen to maltose, maltotriose, and α-dextrines, which are substrates for *Lactobacillus* to produce lactic acid. This leads to a low vaginal pH, which is conducive to the growth of *Lactobacillus* itself. This acidic environment is also essential for the other protective effects of *Lactobacillus*, such as antimicrobial activity and anti-inflammatory effects [61].

Apart from *Lactobacillus*, members of the phylum Actinobacteria were also frequently reported, especially *Gardnerella, Bifidobacterium,* and *Atopobium* although they only make up a small part of the microbiome. *Bifidobacterium* is another bacteria genus that might play an important role in the female genital tract. Similar to *Lactobacillus,* they too confer protection by producing lactic acid and hydrogen peroxide. This prevents the overgrowth of harmful bacteria and helps to maintain the homeostasis of the vaginal microbiome [62]. *Gardnerella* and *Atopobium*, on the other hand, are frequently associated with bacterial vaginosis (BV), which is the most prevalent bacterial vaginal infection in women of reproductive age. Although both microorganisms are usually detected as a component of the vaginal microbiome in women with BV, it has been found that the involvement of *Atopobium* in BV rarely occurs in the absence of *Gardnerella*. Therefore, it is hypothesised that *Atopobium* benefits from *Gardnerella* to survive [63]. *Prevotella,* a Gram-negative anaerobic bacteria under the phylum Bacteroidetes, is also associated with BV. Similarly, there is also a symbiotic relationship between *Gardnerella* and *Prevotella*, whereby the presence of either stimulates the growth of the other [64]. From the phylum Fusobacteria, *Sneathia* was the only genus identified. This genus of Gram-negative, anaerobic bacteria is also postulated to be involved in the pathogenesis of BV [65]. While these BV-associated organisms exist in the vagina alongside *Lactobacillus*, they are kept dormant by the protective actions of *Lactobacillus* as stated earlier. For these reasons, the vaginal microbiota in healthy women would be expected to exhibit lactobacilli dominance [61].

However, the mean relative abundance of *Lactobacillus* identified in the vagina has a wide range. Kyono et al. [19] found that 99.80% of the vagina was composed of *Lactobacillus*, but Lin et al. [36] documented that the abundance of *Lactobacillus* was only 43.88%. This can be due to patient characteristics in the latter study. The healthy controls were negative for BV based on the guidelines of the Infection Disease Society of America, but from the viewpoint of traditional Chinese medicine (TCM), they were classified into either having spleen-deficiency syndrome or damp-heat syndrome. In these classifications, patients displayed distinct symptoms such as leucorrhea and tongue coating, as observed in TCM examinations [36]. The correlation between these syndromes and Western medicine diagnoses is unclear, thus its effect on the vaginal microbiome is unknown.

*Lactobacillus* was also the most abundant taxon in the cervix of healthy, fertile women, ranging from 64.3% to 96.2%. However, no studies with paired samples from the vagina and cervix compared the abundance of *Lactobacillus* between both sites. Other bacteria that were identified in the cervix include *Gardnerella, Bifidobacterium, Atopobium, Prevotella*, and *Sneathia*. The anatomical continuity can explain the similarity in the microbiome of the vagina and cervix although the cervix is considered a part of the upper genital tract.

Several studies on the microbiome of the endometrium were found. However, only three studies were included in this review by applying the exclusion criteria. There is also a discrepancy between the results of different studies. Moreno et al. [18] and Kyono et al. [19] collected endometrial fluid through aspiration and reported that *Lactobacillus* dominated the endometrial microbiome. On the other hand, Fang et al. [17] used endometrial swabs and noted that the abundance of *Lactobacillus* was 6.23%. All three studies also assessed the vaginal microbiota, and the first two revealed that the endometrium and vagina shared similar microbial community composition, while Fang et al. found that the microbial population in the endometrium was quite different from that in the vagina. It is unclear whether this difference is due to different sample collection techniques. In all the sampling procedures, the cervix was first disinfected. Endometrial fluid was aspirated through a trans-cervical catheter, whereas endometrial swabs with sleeves were inserted into the uterine cavity. In both methods, care was taken to avoid contact with the vaginal wall to minimize the risk of contamination. Nevertheless, as sampling was done through the trans-cervical route, there was still a chance of cross-contamination with the cervical microbiota. This makes it hard to ascertain whether *Lactobacillus* identified in the endometrium ascended from the vagina or if they are true colonisers of the uterine cavity. Samples collected through laparoscopy, laparotomy, or hysterectomy would eliminate this problem. However, gaining consent for this to be carried out in healthy, fertile women is impossible. Therefore, no consensus exists regarding the healthy bacterial microbiome configuration in the endometrium.

To date, there are comparatively few studies assessing the microbiome of the fallopian tube, and only two studies were included in this review. In the study conducted by Pelzer et al. [15], some patients were prescribed oral tinidazole in the evening before surgery. Although antibiotic use was not listed as the exclusion criteria, it can potentially alter the microbiome of the fallopian tube. Tinidazole has antimicrobial actions and is active against protozoa and obligate anaerobic bacteria. Therefore, anaerobes might be under-represented in women who received tinidazole. The study by Zhou et al. (2019) only provided data on phyla level and found that Proteobacteria was the most abundant. Proteobacteria are the largest phylum within the bacteria domain, but other than the common trait of being Gram-negative, no specific morphological or physiological traits characterise the members within each class [66]. As the results from the study are non-specific, they only contribute minimally to our understanding of the microbiome of the fallopian tube.

Despite the lack of studies, it is obvious that the microbiome of the lower genital tract differs significantly from the upper genital tract, with the endometrium likely being a zone of transition. Contrary to the previous belief that the upper genital tract is sterile, it actually harbours its own resident microbiota and represents a distinct ecological niche compared to the lower genital tract [9, 15]. Overall, a trend can be observed along the female genital tract. *Lactobacillus* is the only genus that was identified in all the genital sites. Its abundance is highest in the vagina, gradually decreasing along the upper genital tract. The difference in pH throughout the female genital tract can explain this. As mentioned above, *Lactobacillus* thrives in an acidic environment. In general, pH levels are lower in the vagina and cervix compared to the uterus and fallopian tube [67]. For this reason, even if lactobacilli ascends into the upper genital tract, it is unlikely to colonise the site due to unsuitable living conditions.

## Tubal Pregnancy and the Genital Tract Microbiome

The fallopian tube is the most common site of ectopic pregnancy; however, no studies conducted were eligible for our scoping study. Nevertheless, studies have postulated several theories on the pathogenesis of ectopic pregnancy. Evolving into an inflammatory environment may potentially be caused by or cause changes in the fallopian tube microbiome [10]. In fact, it is important to note that the fallopian tube microbiome is especially different from the vagina and cervix, which are *Lactobacillus*-dominant. Therefore, the suitability of the lower genital tract microbiome as a proxy for resembling the fallopian tube condition, microbiota profile, and microenvironment is still a topic for discussion. Currently, insufficient evidence exists regarding potential associations between the microbiome of the upper and lower genital tracts. Few studies have demonstrated a shift from the microbiome of the upper genital tract to that of the lower genital tract, or *vice versa*. In a recent study, researchers conducted a nested case-control study comparing the vaginal microbiome of women with fallopian tube pregnancy and intrauterine pregnancy in the first trimester. Changes in relative abundances of various taxa were identified in women with fallopian tube pregnancy; specifically, genus *Gardnerella*, genus *Prevotella*, class Clostridia, and family Leptotrichiaceae were significantly increased. In contrast, there were no significant changes in the relative abundance of *Lactobacillus* [47]. The justification for researching the correlation between the microbiomes of the upper and lower genital tracts stems from the practical advantage and feasibility of obtaining samples from the lower genital tract. This is particularly significant if a potential proxy can be identified and utilised as a screening or diagnostic biomarker for reproductive conditions in the future. The exploration of the genital microbiome may pave the way for innovative approaches to reproductive health assessment, offering valuable insights and opportunities for enhanced diagnostics and interventions.

## Clinically Significant Pathogens in Ectopic Pregnancy

No doubt, a lot of focus and attention has been given to the *Lactobacillus* genus and its different species by researchers, as it is the dominant taxa in the lower genital tract of the majority of women. However, it is crucial to note that the various microorganisms do not function individually but instead work as a system. Consequently, it is not only the *Lactobacillus* genus that matters. Bacteria that are present in minute amounts or very small relative abundances may have great effects or clinical significance. Such observations have been widely reported in other human microbiome research, such as the oral [68] and gut microbiome [69, 70]. A pattern noted is that the “causative organism” in a disease, which is usually cultured or detected by PCR, is not actually present in high relative abundances. For example, a high abundance of genus *Chlamydia* may be expected in *Chlamydia trachomatis* (CT) infection, but this is not the case. Several studies have shown relative abundances of *Chlamydia* of less than 0.1% in both the vaginal and cervical samples [21, 34, 56]. Various studies have described the strong associations between prior CT infections with ectopic pregnancy, where tubal damage was one of the potential mechanisms underlying this correlation [71, 72]. A recent study assessing the presence of *chlamydia* IgG in women with a confirmed diagnosis of ectopic pregnancy showed that the odds for *chlamydia* infection were higher compared to normal pregnancies [73]. Additionally, the majority of the cases from this study did not have the classic risk factors associated with ectopic pregnancy which further ascertained the need to explore female reproductive tract dysbiosis as a potential cause.

## Current Limitations

There are some limitations to this review. First of all, the number of studies was insufficient, especially those from the upper genital tract, due to the technical and ethical difficulties. Most vaginal samples were collected through a vaginal swab, which is simple to perform. In contrast, fallopian tube samples were collected through dissection following procedures involving salpingectomy, which is invasive. Because of this, it is also harder to recruit healthy subjects other than women who were undergoing salpingectomy for benign conditions, but the effects of these conditions on the microbiome are unknown.

Although all studies utilised next-generation sequencing techniques, they varied in their selection of hypervariable regions to explore the microbiota of the female genital tract, which is a critical factor that significantly influences the depth and precision of microbial community analysis. Sirichoat et al. compared the taxa identified by sequencing the V2, V3, V4, V6-7, V8, and V9 regions of the 16S rRNA gene separately. It was found that each individual region could uniquely identify bacteria taxa that were not identified by other regions. For example, *Brevibacterium, Finegoldia, Ruminococcus*, and *Howardella* were only detected by V3 and not the other hypervariable regions although these genera are not significant in regards to the microbiome of the female genital tract [45]. The regions also differed in the number of taxa identified, with the highest being V3, followed by V6-7, V4, V2, V8, and V9. Besides, the same study also found that the results generated by V3 were the most similar compared to those obtained when all regions were sequenced, implying that V3 would be the most accurate representation of the microbiome of the female genital tract [45]. This is another potential cause of incongruence, and the adoption of standardised methodology will facilitate comparison between studies. Furthermore, when handling low biomass samples such as fallopian tubes and endometrial fluid, a negative control should be included in the studies in order to remove potential laboratory contaminants.

In this review, there were a few exclusion criteria for the characteristics of the patients included in the individual studies. Postmenopausal status, use of hormonal contraception, and pregnancy are factors that might change the milieu of the female genital tract, and studies that recruited these patients were excluded. However, some studies did not specify whether the subjects were pre- or post-menopausal, or whether they used hormonal contraception. These studies were included nevertheless but might provide a different result from the other studies that controlled for these parameters.

In conclusion, a general trend in changes in the microbiome profile has been noted, with mainly a reduction of *Lactobacillus* and an increase in other anaerobic bacteria in the lower genital tract in the disease state. Changes in the upper genital tract are inconclusive and future research with a standardised methodology addressing limitations in our current review can be conducted to determine changes with greater confidence. Researchers should also investigate minor taxa in various reproductive health conditions for their clinical significance. To reiterate, more studies with larger sample sizes, longitudinal studies, collection of data on patient characteristics, standardised sequencing platforms, and hypervariable regions should be considered in the future to achieve valid and convincing results. Finally, microbial metabolomics or shotgun metagenomics [74] can be performed in order to explore functional relationships of the female genital tract microbiota. It will be of immense clinical significance if a proxy can be found.
