# Supplementary material for: A Landscape View of the Female Genital Tract Microbiome in Healthy Controls and Women With Reproductive Health Conditions Associated With Ectopic Pregnancy
Source: Br J Biomed Sci. 2024 Jan 12;80:12098. doi: 10.3389/bjbs.2023.12098 (PMC10811206; doi:10.3389/bjbs.2023.12098)
Supplement: Supplementary file 1 [file Table1.docx]

**Supplementary Data 1: Study characteristics of literature included for female genital tract with reproductive conditions.**

| Study | Disease | Sampling | Sequencing | | Cohort | |
| --- | --- | --- | --- | --- | --- | --- |
|  |  | **Sample type/ location** | **Sequencing platform** | **Hypervariable region** | **Sample size** | **Country** |
| Amato et al. (2020) ^2^ | IUI failure | Vaginal swab | MiSeq (Illumina) | V3-V4 | 23 | Italy |
| Ata et al. (2019) ^3^ | Stage 3-4 endometriosis | Endo-cervical swab | MiSeq (Illumina) | V3-V4 | 14 | Turkey |
| Bernabeu et al. (2019) ^4^ | ART failure | Vaginal swab | MiSeq (Illumina) | V3-V4 | 17 | Spain |
| Campisciano et al. (2017) ^5^ | Idiopathic infertility | Vaginal swab | Ion PGM sequencing 200 (Thermo Fisher) | V1-V3 | 14 | Italy |
|  | Diagnosed infertility | Vaginal swab | Ion PGM sequencing 200 (Thermo Fisher) | V1-V3 | 13 | Italy |
|  | Vaginosis | Vaginal swab | Ion PGM sequencing 200 (Thermo Fisher) | V1-V3 | 39 | Italy |
| Ceccarani et al. (2019) ^6^ | Bacterial vaginosis | Vaginal swab | MiSeq (Illumina) | V3-V4 | 20 | Italy |
|  | Chlamydia trachomatis | Vaginal swab | MiSeq (Illumina) | V3-V4 | 20 | Italy |
|  | Vulvovaginal candidiasis | Vaginal swab | MiSeq (Illumina) | V3-V4 | 18 | Italy |
| Cregger et al. (2017) ^7^ | Stage 3 endometriosis | Cervical swab | MiSeq (Illumina) | V3-V5 | 1 | US |
|  | Endometriosis - other stages | Cervical swab | MiSeq (Illumina) | V3-V5 | 9 | US |

**Supplementary Data 1**: cont.

| Study | Disease | Sampling | Sequencing | | Cohort | |
| --- | --- | --- | --- | --- | --- | --- |
|  |  | **Sample type/ location** | **Sequencing platform** | **Hypervariable region** | **Sample size** | **Country** |
| Di Pietro et al. (2018) ^8^ | Chlamydia trachomatis | Endo-cervical swab | MiSeq (Illumina) | V3-V4 | 7 | Italy |
|  | HPV/ CT coinfection | Endo-cervical swab | MiSeq (Illumina) | V3-V4 | 3 | Italy |
|  | HPV | Endo-cervical swab | MiSeq (Illumina) | V3-V4 | 8 | Italy |
| Fang et al. (2016) ^9^ | Endometrial polyps | Endometrial swab | MiSeq 250 (Illumina) | V4 | 10 | China |
|  | Endometrial polyps/ Chronic endometritis | Endometrial swab | MiSeq 250 (Illumina) | V4 | 10 | China |
| Filardo et al. (2017) ^10^ | Asymptomatic Chlamydia trachomatis | Endo-cervical swab | MiSeq (Illumina) | V3-V4 | 10 | Italy |
| Filardo et al. (2019) ^11^ | Chlamydia trachomatis | Endo-cervical swab | MiSeq (Illumina) | V4 | 39 | Italy |
| Graspeuntner et al. (2018) ^12^ | Infectious infertility | Cervical swab | MiSeq (Illumina) | V3-V4 | 21 | Germany |
|  | Non-infectious infertility | Cervical swab | MiSeq (Illumina) | V3-V4 | 26 | Germany |
| Hernandes et al. (2020) ^13^ | Deep endometriosis | Vaginal swab | MiSeq (Illumina) | V3-V4 | 10 | Brazil |
|  | Deep endometriosis | Endometrial tissue | MiSeq (Illumina) | V3-V4 | 18 | Brazil |

**Supplementary Data 1**: cont.

| Study | Disease | Sampling | Sequencing | | Cohort | |
| --- | --- | --- | --- | --- | --- | --- |
|  |  | **Sample type/ location** | **Sequencing platform** | **Hypervariable region** | **Sample size** | **Country** |
| Hong et al. (2016) ^14^ | Bacterial vaginosis | Vaginal swab | 454 pyrosequencing (Roche) | V3-V5 | 12 | Korea |
| Hong et al. (2021) ^15^ | PCOS | Vaginal swab | HiSeq 2500 (Illumina) | V3-V4 | 89 | China |
| Khan et al. (2016) ^16^ | Endometriosis | Endometrial swab | MiSeq (Illumina) | *unknown* | 16 | Japan |
| Kong et al. (2020) ^17^ | IVF failure | Vaginal swab | HiSeq 2000 (Illumina) | V4 | 21 | China |
| Kwasniewski et al. (2018) ^18^ | HPV infection - LSIL | Cervical swab | *unknown* | V4 | 95 | Poland |
|  | HPV infection - HSIL | Cervical swab | *unknown* | V4 | 85 | Poland |
| Liu et al. (2021) ^19^ | Empty-sac miscarriage | Vaginal swab | MiSeq (Illumina) | V4 | 13 | China |
|  | Missed miscarriage | Vaginal swab | MiSeq (Illumina) | V4 | 22 | China |
| Liu et al. (2019) ^20^ | Infertility without chronic endometritis | Endometrial fluid | MiSeq (Illumina) | V4 | 118 | China |
| Liu et al. (2019) ^21^ | Intrauterine adhesion | Vaginal swab | HiSeq 2000 (Illumina) | V4 | 50 | China |
| Lozano et al. (2021) ^22^ | Chronic endometritis | Vaginal swab | MiSeq (Illumina) | V3-V4 | 30 | Spain |
|  | Chronic endometritis | Endometrial swab | MiSeq (Illumina) | V3-V4 | 30 | Spain |

**Supplementary Data 1:** cont.

| Study | Disease | Sampling | Sequencing | | Cohort | |
| --- | --- | --- | --- | --- | --- | --- |
|  |  | **Sample type/ location** | **Sequencing platform** | **Hypervariable region** | **Sample size** | **Country** |
| Moreno et al. (2016) ^23^ | Failure of implantation | Endometrial fluid | 454 pyrosequencing (Roche) | V3-V5 | 15 | Spain |
|  | Miscarriage in infertile women | Endometrial fluid | 454 pyrosequencing (Roche) | V3-V5 | 5 | Spain |
| Riganelli et al. (2020) ^24^ | Infertility | Vaginal swab | MiSeq (Illumina) | V3-V4 | 30 | Italy |
|  | Infertility | Endometrial fluid | MiSeq (Illumina) | V3-V4 | 30 | Italy |
| Ruan et al. (2021) ^25^ | Tubal pregnancy | Vaginal swab | MiSeq (Illumina) | V3-V4 | 32 | China |
| Tu et al. (2020) ^26^ | PCOS | Vaginal swab | MiSeq (Illumina) | V3-V4 | 47 | China |
|  | PCOS | Cervical swab | MiSeq (Illumina) | V3-V4 | 47 | China |
| Vladislavovna et al. (2020) ^27^ | Infertility | Endometrial fluid | MiSeq (Illumina) | V3-V4 | 22 | Russia |
| Wang et al. (2020) ^28^ | Aerobic vaginitis | Vaginal swab | HiSeq2500 (Illumina) | V4 | 80 | China |
| Xu et al. (2020) ^29^ | Embryonic miscarriage | Vaginal swab | HiSeq3000/4000 (Illumina) | V4 | 25 | China |
| Zhao et al. (2020) ^30^ | Infertility | Vaginal swab | HiSeq 2500 (Illumina) | V1-V2 | 30 | China |
| Zhou et al. (2019) ^31^ | LR-HPV Infection | Vaginal swab | MiSeq (Illumina) | V3-V4 | 42 | China |
